# Human amnion-derived mesenchymal stem cells improve subclinical hypothyroidism by immunocompetence mediating apoptosis inhibition on thyroid cells in aged mice

**DOI:** 10.1007/s00441-023-03822-1

**Published:** 2023-08-12

**Authors:** Chuyu Li, Qiang Rui, Xiaohan Dong, Song Ning, Jing Zhou, Huimin Wu, Chunyan Jiang, Yugui Cui, Jiayin Liu, Jun Jiang, Lianju Qin

**Affiliations:** 1 State Key Laboratory of Reproductive Medicine, Center of Clinical Reproductive Medicine, The First Affiliated Hospital of Nanjing Medical University, Jiangsu Province Hospital, Nanjing, 210029 China; 2 Department of General Surgery, Jiangsu Province Hospital of Chinese Medicine, Affiliated Hospital of Nanjing University of Chinese Medicine, Nanjing, 210000 China; 3 Department of General Surgery, The First Affiliated Hospital of Nanjing Medical University, Jiangsu Province Hospital, Nanjing, 210029 China

**Keywords:** Human amniotic mesenchymal stem cells (hAMSCs), Subclinical hypothyroidism, Aging, Immune regulation, Apoptosis

## Abstract

Subclinical hypothyroidism (SCH) affects 10% of the global population, which is most prevalent in women and the elderly. However, it remains debatable whether the elderly with subclinical hypothyroidism needs thyroxine supplement. Human amnion-derived mesenchymal stem cells (hAMSCs) could play important roles in autoimmune diseases, suggesting that hAMSC be a candidate to regulate the thyroid function of female age-related subclinical hypothyroidism. Herein, we established the model of SCH in the aged female mice. This study was designed to investigate whether human amnion-derived mesenchymal stem cells (hAMSC) could effect on immune regulation, apoptosis inhibition of thyroid cells, thyroid function, blood lipid levels, and heart function. In addition, qualified hAMSCs were intravenously injected into aged female SCH mice via the tail vein on day 0 and day 10. The levels of thyroid hormone and blood lipids as well as cardiac function, serum immunological indexes, and apoptosis of thyroid cells were then analyzed on day 5, 10, 15, and 20; meanwhile, the quantity of Th1, Th2, Th17, and Treg immune cells in peripheral blood was evaluated before and on day 20 post-injection. Our study demonstrated that after hAMSC transplantation, the thyroid functions, blood lipid levels, and heart function indexes of age-related SCH (AR-SCH) mice were significantly improved. Consistent with this, Th1 and Treg cells increased significantly, while Th2 and Th17 cells decreased in peripheral blood. Apoptosis was also suppressed in the thyroid cells. In summary, hAMSC delivery can potentially be a safe and effective therapy for treating SCH in the elderly, improving related complications by immunomodulatory and apoptosis inhibition.

**Supplementary Information:**

The online version contains supplementary material available at 10.1007/s00441-023-03822-1.

## Introduction

Subclinical hypothyroidism (SCH) is an endocrine and metabolic disease in which the level of serum thyroid-stimulating hormone (TSH) is high, but that of serum-free thyroxine (FT4) is normal (Biondi et al. 2019). Hence, elevated amounts of thyroid autoantibodies, especially those of thyroglobulin and anti-thyroid peroxidase, can be used as a marker for diagnosing the condition (Vanderpump et al. 1995). Regarding its etiology, SCH may occur as a result of various diseases that damage the thyroid’s structure or impact the normal synthesis of thyroid hormones (Jones et al. 2010).

The incidence of SCH is 3–8% in the general adult population (Hennessey et al. 2015), but it may reach 20% in the elderly due to the natural aging process that causes alterations in the anatomy and activities of the thyroid gland (Baskin et al. 2002). Additionally, sex is also a risk factor for SCH, with females having a three- to five-fold increased risk compared to males (Vanderpump 2011). These two factors (i.e., age and sex) further explain why the likelihood of SCH in women over 60 years is 20% higher in comparison to men of the same age. According to the extent of thyroid deficiency, hypothyroidism may range in severity (Laurberg et al. 2005). Nonetheless, the condition is often identified by a range of chemical and clinical anomalies which increase the probability of cardiovascular diseases (Mariotti et al. 2007). In fact, once SCH diagnosed, certain conditions such as abnormal dyslipidemia, especially the increase of total cholesterol(T-CHO) and low-density lipoproteins (LDL-C), can further help to assess whether additional treatment would be required (Asvold et al. 2007).

Untreated SCH can lead to adverse consequences according to guidelines from endocrinology societies (Pearce et al. 2013). Treatment is often achieved using L-thyroxine﻿ (Rodriguez-Gutierrez et al. 2017). However, it has been argued that there are no signs of significant improvement in hypothyroid symptoms when L-thyroxine is used among adults with SCH (Effraimidis et al. 2021).

**Fig. 1:**
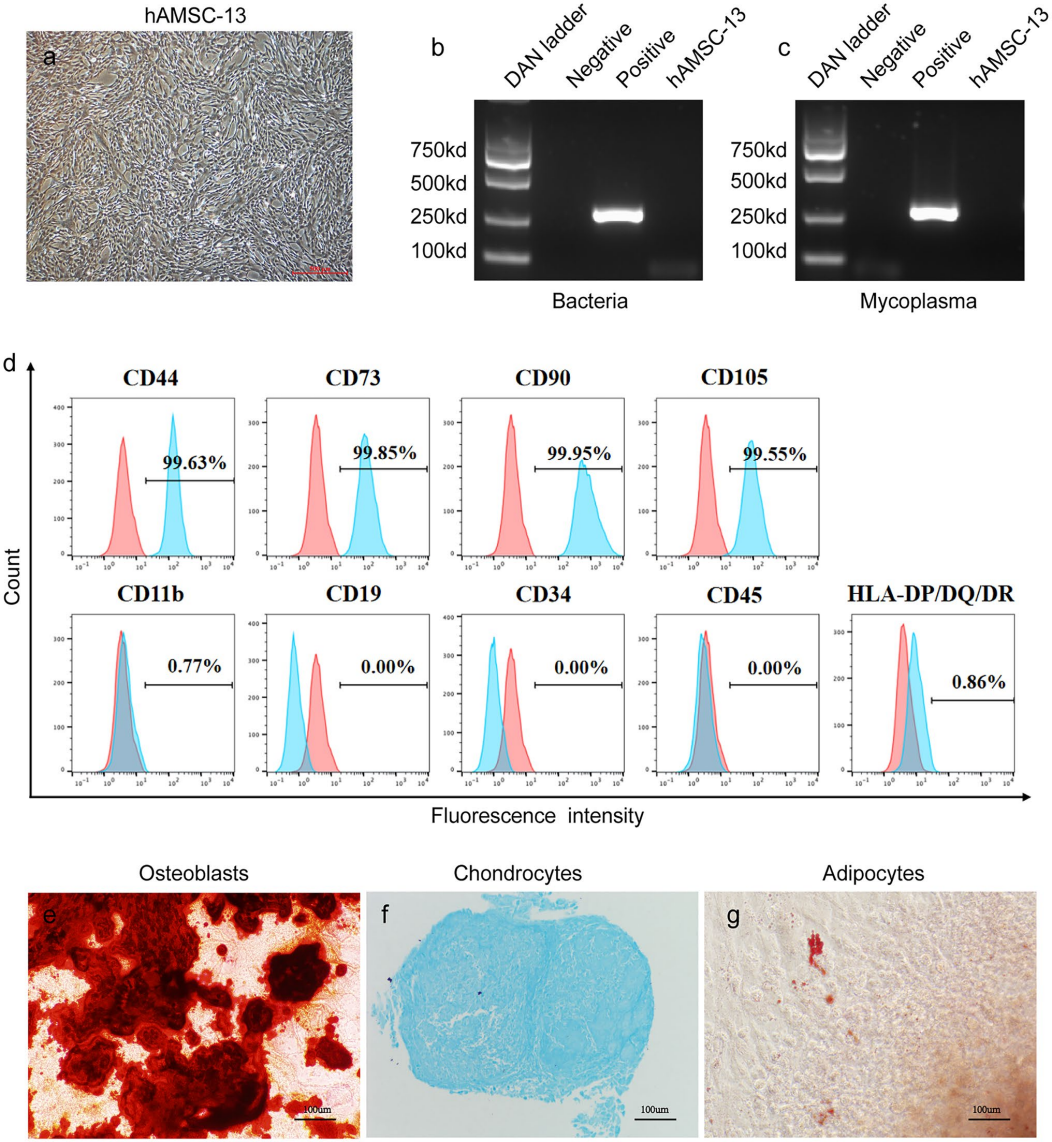
Phenotypic characterization and quality control on hAMSCs. **a** Microscopic images represent the morphology of hAMSCs. Scale bar: 500 μm. **b**, **c** The hAMSC bacteria contamination and mycoplasma infection were tested by real-time fluorescence quantitative polymerase chain reaction. **d** Representative flow cytometry histograms of the expression of hAMSC-specific surface makers. **e**–**g** Differentiation abilities of cells were detected by cellular staining. Cultured hAMSCs could differentiate into osteoblasts (**e**), chondrocytes (**f**), and adipocytes (**g**). Scale bar: 100 μm

**Fig. 2:**
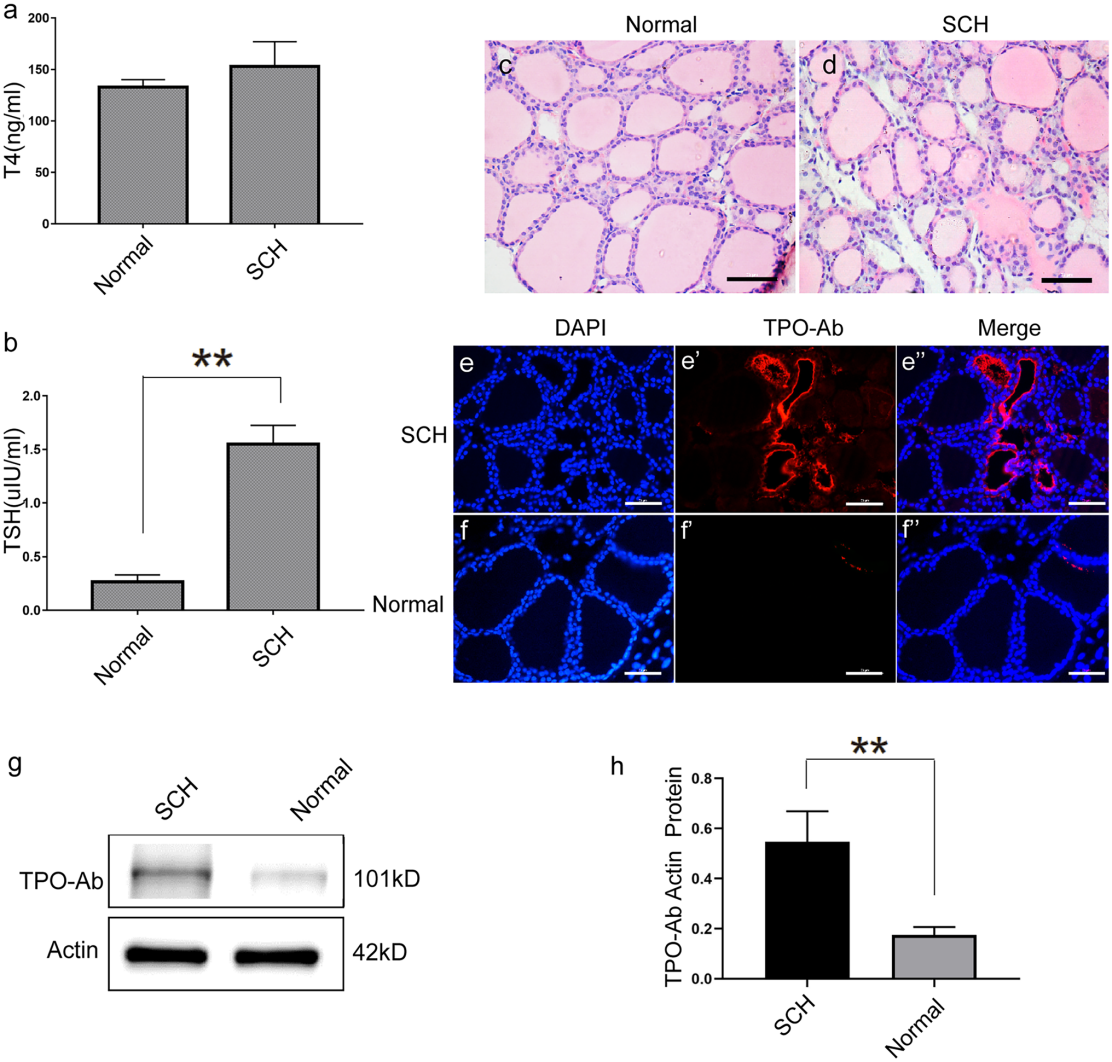
Establishment of age-related subclinical hypothyroidism (AR-SCH) mouse model. **a**, **b** ELISA analysis of the lever of T4 and TSH (*n* = 10, per group, *P* < 0.01, 2-tailed *t*-test).** c**, **d** Histological analysis of paraffin-embedded sections and hematoxylin and eosin (HE) staining on mouse thyroid. Scale bar: 50 μm.** e**–**f’’** Immunofluorescence staining of TPO-Ab in the thyroid.** g**, **h** Western blot analysis of TPO-Ab in thyroid tissues (*n* = 10, per group, error bars represent ± SEM, *P < 0.05, **P < 0.01, ***P < 0.001, 2-tailed* t*-test)

**Fig. 3:**
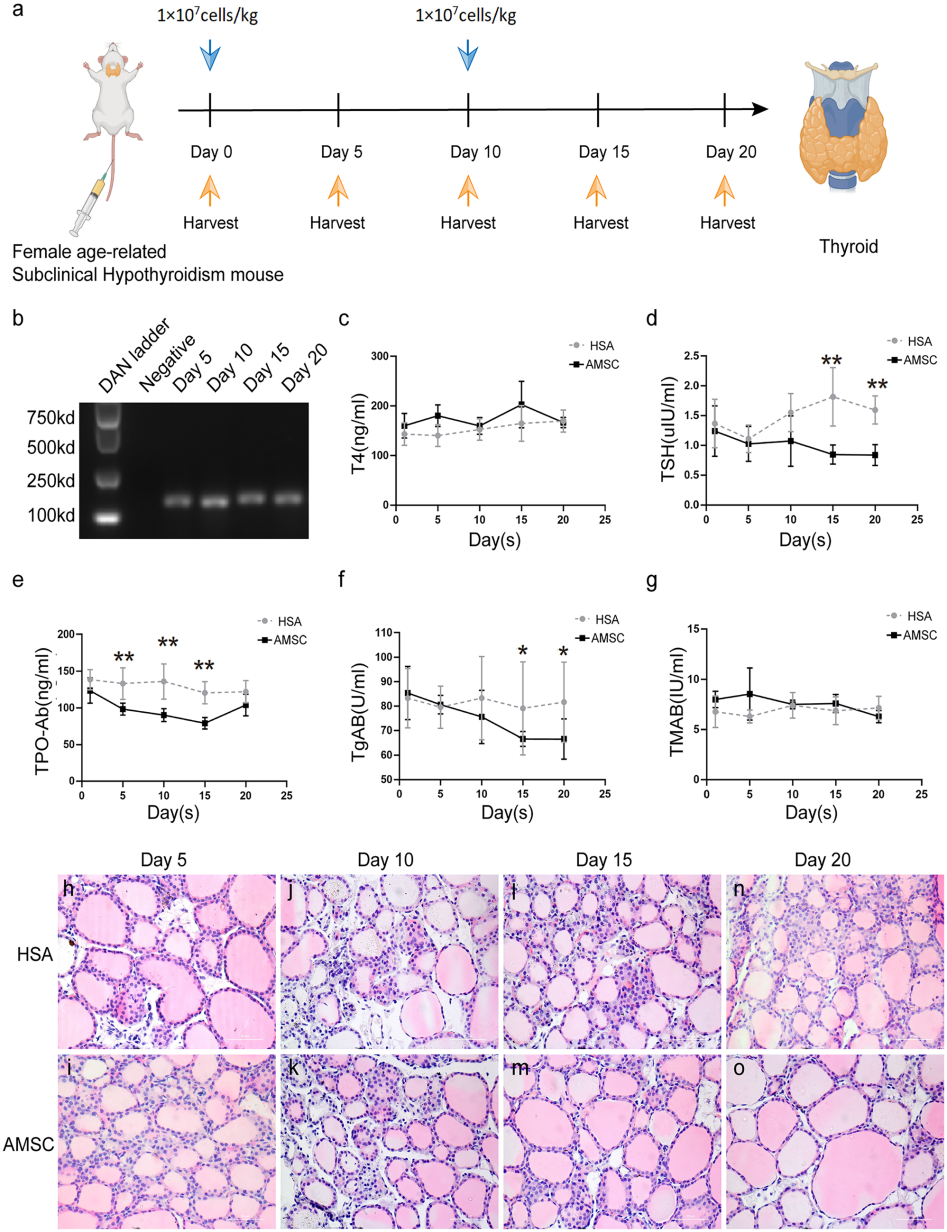
hAMSC homing and effects of cells treatment on biochemical indexes of AR-SCH mouse. **a** The schematic representation of female AR-SCH mouse treatment from day 0 to day 20. **b** Tracing of hAMSCs in thyroid tissues. **c** Serum-free thyroxine (FT4).** d** Serum thyroid-stimulating hormone (TSH). **e** Thyroid peroxidase (TPO). **f** Anti-thyroglobulin antibodies (TGAb). **g** Anti-thyroid microsomes (TMAb). All tests were performed a minimum of three times (*n* = 6, per group, error bars represent ± SEM, *P < 0.05, **P < 0.01, ***P < 0.001, 2-tailed *t*-test). **h**–**o** Histopathology analysis of thyroid tissues from AR-SCH mouse v after treatment. Scale bar: 50 μm

**Fig. 4:**
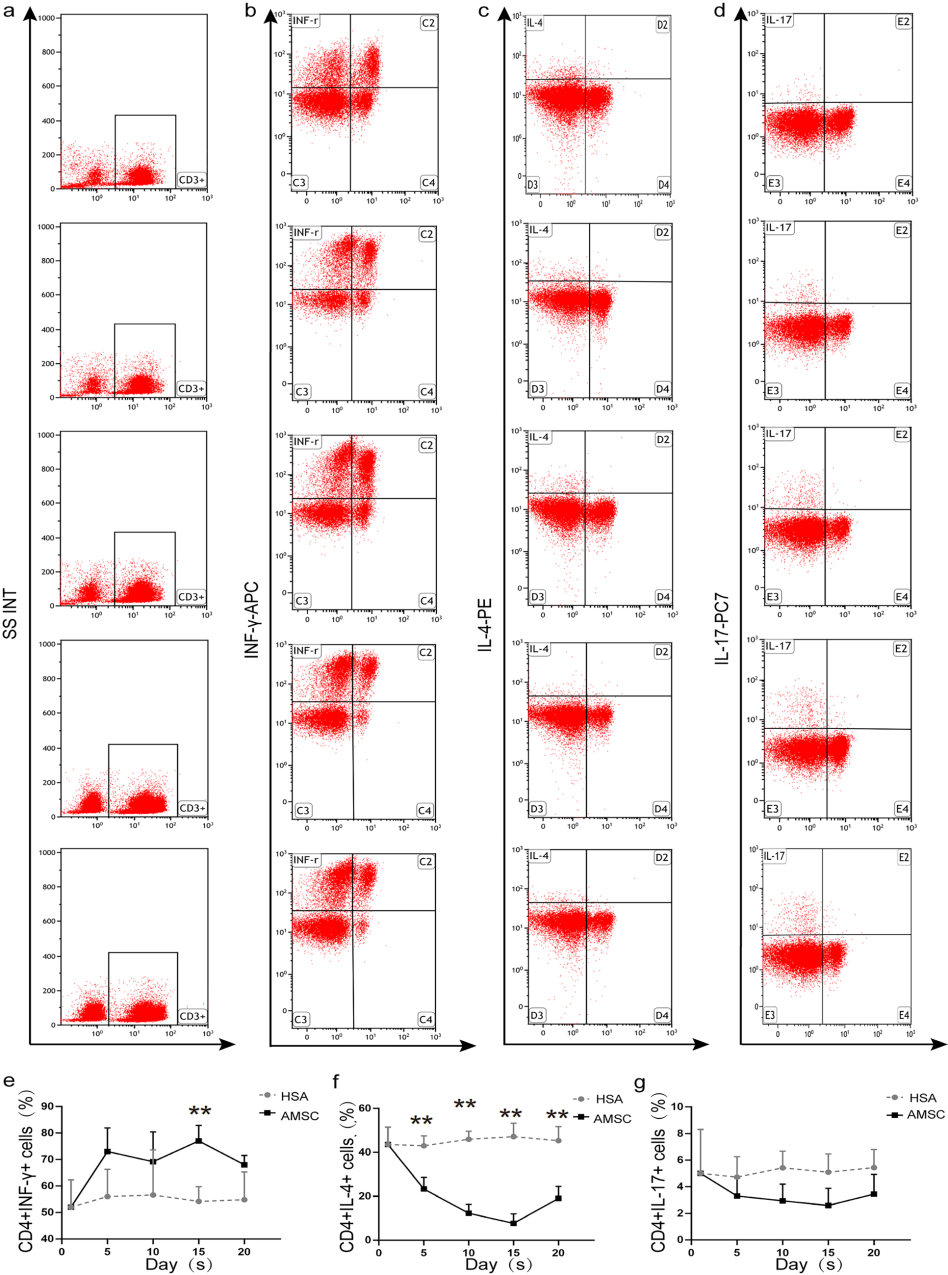
Expression of Th1/Th2/Th17 cell numbers following hAMSC transplantation. **a**–**d** Measure IFN-γ (**b**), IL-4 (**c**), and IL-17 (**d**) expression by flow cytometry. **e**–**g** Quantitative analysis on the release of Th1 cytokine (IFN-γ) (**e**), Th2 cytokine (IL-4) (**f**), and Th17 cytokine (IL-17) (**g**) (*n* = 6, per group, error bars represent ± SEM, *P < 0.05, **P < 0.01, ***P < 0.001, 2-tailed *t*-test). IFN-γ γ-interferon, IL-4 interleukin-4, IL-17 interleukin-17

**Fig. 5:**
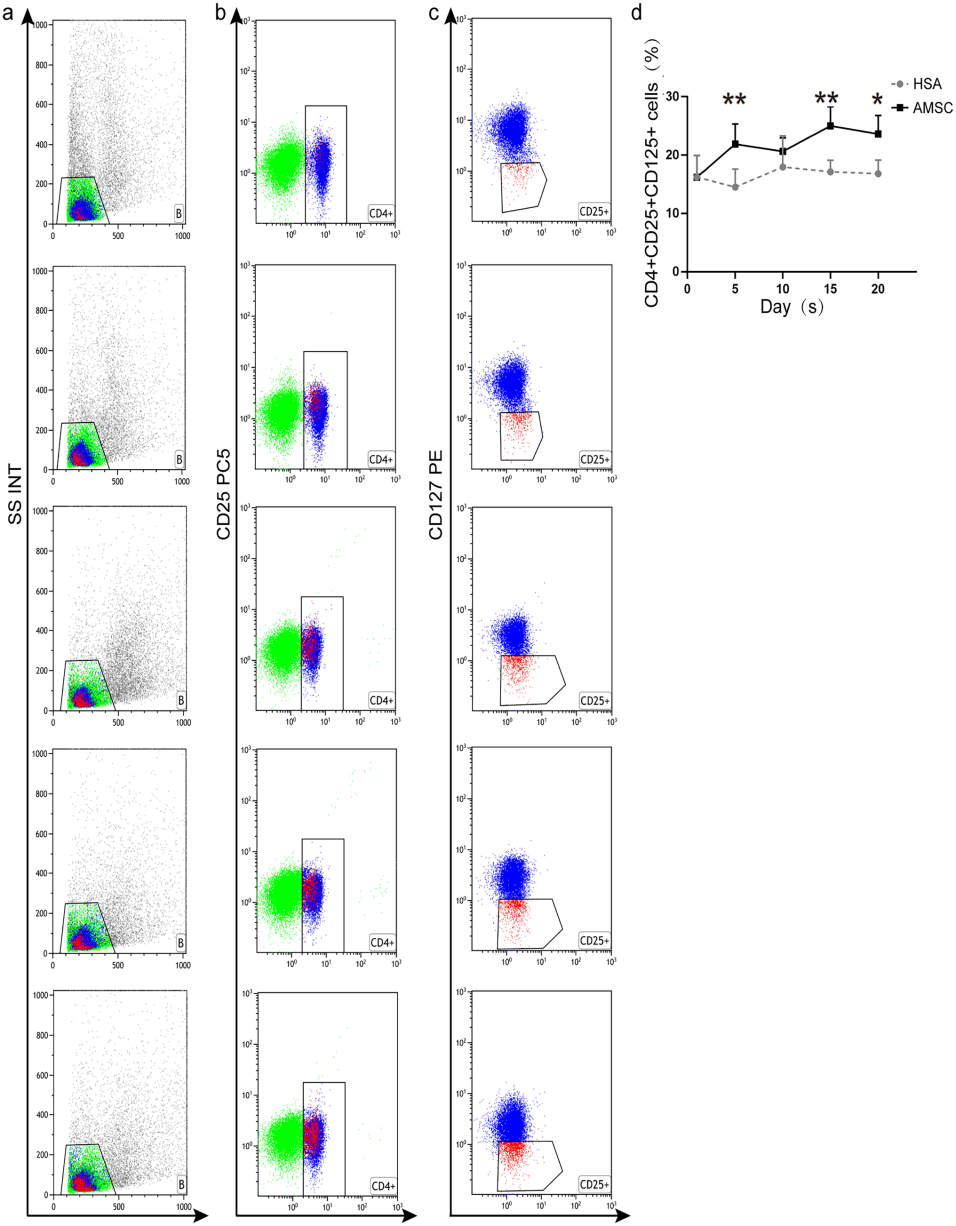
Expression of Treg cell numbers following hAMSC transplantation. **a**–**c** Measure CD25 (**b**) and CD125 (**c**) expression by flow cytometry. **d** Quantitative analysis on the release of Treg cell cytokine (CD25 + CD125 +) (*n* = 6, per group, error bars represent ± SEM, *P < 0.05, **P < 0.01, ***P < 0.001, 2-tailed *t*-test). Treg cell regulatory T cells

**Fig. 6:**
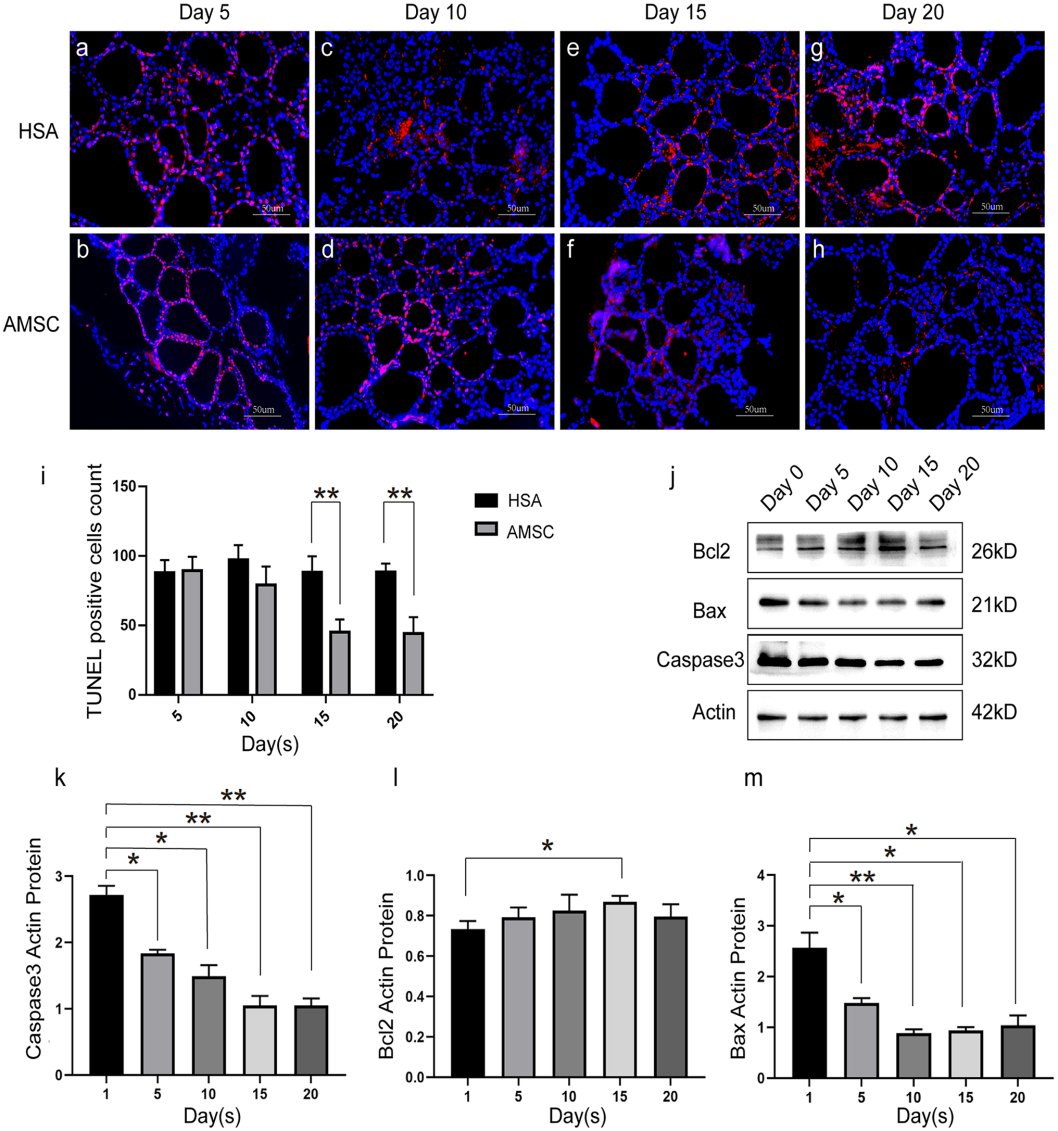
HAMSC treatment alleviated the apoptosis of thyroid tissue in the AR-SCH mice. **a**–**i** TUNEL analysis of the number of apoptosis cells in thyroid tissues. **j**–**m** Western blot determined the Bcl-2 (**l**), BAX (**m**), and caspase3 (**k**) expression levels of proteins in mouse thyroid. All tests were performed three times (*n* = 6, per group, error bars represent ± SEM, *P < 0.05, **P < 0.01, ***P < 0.001, 2-tailed *t*-test)

**Table 1:** Primers used in the study

Primer name	Sequence (5ʹ-3ʹ)
Bacterial-F	CAGCMGCCGCGGTAATWC
Bacterial-R	GGACTACCAGGGTATCTAAT
Mycoplasma-F	GGGAGCAAACAGGATTAGATACCCT
Mycoplasma-R	TGCACCATCTGTCACTCTGTTAACCTC
SRY-609F	CCCGAATTCGACAATGCAATCATATGCTTCTGC
SRY-609R	CTGTAGCGGTCCCGTTGCTGCGGTG

The pathogenesis of SCH is complex, but it closely related to the immune dysfunction of thyroid tissue (Almandoz et al. 2012). Therefore, regulating and restoring the body’s autoimmune function is the core and key to the treatment of SCH. It has been postulated that cellular therapy based on immunomodulatory mesenchymal stem cells (MSC) might be an effective treatment for SCH. Indeed, human amniotic mesenchymal stem cells (hAMSCs) were reported to be promising to protect tissues or organs by immunoregulation in addition to regulating angiogenesis anti‐inflammatory, and antiapoptosis (Liu et al. 2014).

Importantly, it has now been demonstrated in several studies in animal models of thyroid disease that MSCs can significantly ameliorate thyroid function (Ma et al. 2017). In murine experimental autoimmune thyroiditis, murine CTLA4Ig gene-transduced human ASCs diminish inflammatory immune response and enhance Th1/Th2 balance (Choi et al. 2015).

So far, there is no report on the immunomodulatory effects of hAMSCs on thyroid illnesses, especially on SCH. In this study, the efficacy of hAMSCs in the treatment of AR-SCH as well as its effects on blood lipid levels and cardiac function was investigated using an AR-SCH model. The therapeutic mechanism was assessed, especially in terms of immunomodulatory and apoptosis inhibition. The results indicated that hAMSC application was effective and feasible on hypothyroidism treatment, providing a possible novel therapy for SCH.

## Materials and methods

### HAMSC isolation and identification

This study was approved by the institutional ethics committee of the First Affiliated Hospital, Nanjing Medical University. Healthy women who had given birth in the Jiangsu Women and Children Hospital were recruited to donate human amniotic membrane, and written informed consent was obtained. HAMSCs were isolated, characterized, and established cell lines as previously described (Qin et al. 2022). Cell line of AMSC13 was employed, and hAMSC (passage number five) was prepared as described previously (Alviano et al. 2007) for application in all transplantation experiments. Prior to transplantations, the cells were subjected to quality control to ensure that they were according to specifications in terms of MSC-specific surface markers expression, cell viability, and free of contamination by mycoplasma or bacteria. Flow cytometry was used to assess the expression of MSC-specific surface markers, and the following monoclonal phycoerythrin-conjugated antibodies were used in the analysis: CD45 (BD, 555,483), CD34 (BD, 555,822), HLA-DP/DQ/DR (BD, 562,008), CD19 (BD, 555,413), CD11b (BD, 555,388), and CD105 (eBioscience, 2–10 (BD, 555,479). That 10,000 cells/antibody was counted and identified by a flow cytometer (Beckmen, NAVIOS). Data collected were then analyzed using the FlowJo software.

### Mouse model of age-related SCH

In the study, female C57BL/6 mice were utilized to establish age-related SCH model. It is reported C57BL/6 mice of 20 months old are considered to be equivalent to 60-year-old humans (Flurkey et al. 2007). A total of 200 mice of 28-week-old were acquired (Beijing Vital River Laboratory Animal Technology Co., Ltd) and reared in the SPF-class animal room at Nanjing Medical University. The mice had unrestricted access to food and water and were subjected to a day/night lighting cycle of 12 h of light and 12 h of dark. All cages, litter, food, and water were sterilized and disinfected before being used for feeding. When the mice reached 20 months old, serum FT4 and TSH were measured by ELISA. The mouse with high TSH and normal FT4 was determined as age-related SCH (AR-SCH) model and 48 as SCH group out of 200 mice were selected.

### Cell transplantation and treatment of AR-SCH models

The AR-SCH mice were randomly assigned to an hAMSC transplantation group and a control one, each with 24 mice. Mice in the first group were injected hMSCs through tail vein on day 0 and day 10 at dosage of 1.0 × 10^7^ cells/kg of body weight in 300 μl of human serum albumin (HSA). According to our previous study, HSA injection had no effect on mice (Liu et al. 2021). Each control mouse was injected 300-μl HSA only on day 0 and day 10. All injected mice lived healthily to the experimental end point.

Six mice of each group were selected randomly to analyze on days 5, 10, 15, and 20 post the injection. The selected mice were anaesthetized with 1% sodium pentobarbital, collected blood (1 ml/mouse) from their medial canthal vein, and then were euthanized. For each mouse, one side of the thyroid gland and along with parts of the liver and heart were collected. Parts of tissues were fixed in 4% paraformaldehyde and prepared for histological and immunohistochemical examinations. The remaining tissues were stored at − 80 °C for subsequent protein and nucleic acid analysis.

### Extraction of RNA and RT-qPCR assay

Total RNAs were extracted from the thyroid tissues using TRIzol Reagent (Life Technologies). Reverse transcription was then followed by RT-qPCR using RNA-Solv Reagent (TaKaRa) according to the manufacturer’s manual. The primers are listed in Table 1. Here, an Applied Biosystem® Co. Ltd. (QuantStudioTM 7 Flex Real-Time PCR System) instrument was used for the amplification procedure. Finally, the 2^−ΔΔ^Ct technique was used to measure gene expression levels, with the endogenous housekeeping gene GAPDH serving as a reference.

### ELISA-based determination of thyroid peroxidase and thyroglobulin antibody levels

Approximately, 1 ml of blood was extracted from the posterior ocular venous plexus of each anesthetized mouse. After the blood samples rested at room temperature for 30 min, the serum was isolated by centrifuging for 15 min at 3000 rpm and then stored at − 80 °C for later hormone analysis. Finally, FT4 (ASB EK6868), TSH (CUSABIO CSB-E05116m), TPO-Ab (mlbio ml002215), TgAB (CUSABIO CSB-E09543m), and TMAb (mlbio ml037679) were measured by ELISA method on a full-wavelength microplate reader (Thermo Scientific, Multiskan GO).

### Biochemical parameters of plasma

Plasma was isolated from the blood collected above and performed biochemical parameters analysis. Total cholesterol (T-CHO A111-1–1), triglycerides (TG A110-1–1), high-density lipoprotein cholesterol (HDL-C A112-1–1), and low-density lipoprotein cholesterol (LDL-C A113-1–1) were ultimately measured in the plasma according to the manufacturer’s instructions (Nanjing Jiancheng Bioengineering Institute).

### Staining of thyroid glands

The thyroid glands were surgically excised (from one side), fixed in 4% neutral-buffered formalin (Sigma-Aldrich), embedded in paraffin, sectioned (5 m thick), and stained with hematoxylin and eosin (Dako, Carpinteria). The complete samples were observed at 200 × magnification using a light microscope (BX53, Olympus).

### Assessing Th1/Th2/Th17 and Treg cells using flow cytometry

Peripheral blood mononuclear cells (PBMCs) were extracted from the blood collected above and Th1/Th2/Th17 and Treg cells. Total lymphocytes were labeled with anti-human CD3 and CD8 antibodies (BioLegend). IFN-γ (Multiscience) antibodies were used to stain the Th1 cells; IL-4 (Multiscience) antibodies were used to marker the Th2 cells; and IL-17A (Multiscience) antibodies were used to identify the Th17 cells; anti-mouse CD4 FITC (eBioscience), anti-mouse CD25 PE (eBioscience), and anti-mouse CD125 PE (eBioscience) antibodies were used to characterize the Treg cells.

The blood was prevent clotted by adding sodium heparin, and then the PBMCs were isolated by Ficoll (sigma) according to previous report (Yin et al. 2015). Then, brefeldin A (BioLegend) was added, and the cells were collected after 5 h. The isolated PBMCs were washed and resuspended in PBS at concentration of 1 × 10^6^ cells/100 µl.

To detect Th1/Th2/Th17 antibody labeling, the cells were then left to spend 30 min in a dish containing antibodies against CD3 and CD8. Then, the cell membrane was disrupted and fixed using fixation buffer (biolegend: 420,801) and Intracellular Staining Permeabilization Wash Buffer (biolegend: 421,002). Finally, the cells were stained with the aforementioned anti-IL4 FITC, anti-IL17, and anti-IFN-γ antibodies with 30 min. Similarly, Treg changes were detected by flow cytometry. Add 4 µl of CD4 and CD125 antibody solution and 2 µl of CD25 antibody solution. The mixture was vortexed and incubated at 4 °C in darkness for 20 min. All cells were detected by flow cytometry.

### Apoptosis assay

TUNEL BrightRed Apoptosis Detection Kit was used for detecting apoptosis of thyroid cells from each group as specified by the manufacturer (Vazyme, A113). All experiments were performed according to the TUNEL procedure of paraffin-embedded tissue sections. Samples were analyzed under a fluorescence microscope (Zeiss AG, Oberkochen, Germany) and observed in BrightRed red fluorescence at 620 ± 20 nm with a standard fluorescence filter device and the blue fluorescence of DAPI at 460 nm. In a double-blind study, apoptotic cells were tallied in tissue sections by two separate technicians.

### Histology and immunofluorescence

After cell transplantation, the expression of human-specific stem121 in the thyroid was analyzed using immunofluorescence to follow hAMSC homing and differentiation. Thyroid, liver, and heart slices were dewaxed and then microwaved in 10 mM sodium citrate (pH 6.0) for 20 min to conduct heat-mediated antigen retrieval (Beyotime, P0083). Mouse anti-human STEM121 antibody (1:100, TAKARA, Y40410) and rabbit anti-TPO antibody (1:100, CST, 12,829) were used as primary antibodies. Following a wash in deionized water and a rinse in PBS, the sections were incubated in 5% goat serum for 60 min before being incubated with the secondary antibodies, which were CoraLite 488-conjugated goat anti-mouse IgG (H + L) (1:100, Invitrogen, A21202) and goat anti-rabbit IgG (H + L) (1:100, Invitrogen, A21202) (1:100, Abcam, ab150078). DNA counter staining was performed using DAPI (Beyotime, C1006). The data were collected using the same confocal microscopy settings and immunostaining method (Nikon, ECLIPSE Ti).

### Protein extraction and western blot

The collected thyroid glands, livers, and hearts from each group were homogenized in ice-cold RIPA lysis buffer (Thermo Fisher, 89,900), which contains a combination of phosphatase and protease inhibitors (Thermo Fisher, 78,443). The samples’ total protein content was determined using the bicinchoninic acid (BCA) method after being centrifuged for 15 min at 15,000 rpm at 4 °C to isolate protein lysates. The proteins were electrophoretically separated on a 10% SDS PAGE gel and then transferred to a PVDF membrane (Bio-Rad, 1,620,177). After, incubate the membranes overnight at 4 °C in the presence of the following primary antibodies: rabbit anti-TPO (1:1000, Abcam, ab278525), anti-human STEM121 antibody (1:100, TAKARA, Y40410), rabbit anti-Bcl-2 (1:1000, Abcam, ab182858), rabbit anti-BAX (1:1000, Abcam, ab182733), rabbit anti-caspase3 (1:1000, Abcam, ab32499), and rabbit anti-actin (1:5000, Proteintech, 23,660–1-AP). Then samples were blocked with 5% no-fat dry milk (1:5000, Proteintech, 10,494–1-AP). Afterwards, the membranes were incubated with horseradish peroxidase (HRP)-conjugated anti-rabbit secondary antibody (1:1000, Abcam, ab6721) for 1 h at room temperature, and proteins were identified using an ECL kit (Thermo Fisher, 32,209). Data was analyzed using ImageJ software (NIH, Bethesda). Each experiment was performed in triplicate with actin serving as the control.

### Echocardiography

After anesthetized with 1.5% isoflurane, mice lying supine were scanned using a 30-MHz linear probe on a Vevo 3100 imaging system (VisualSonics, Canada). The inside temperature was maintained at 37 °C. The parasternal long-axis view was used for the first 2D echocardiographic evaluation of LV size and systolic performance. The quality was maximized by adjusting the image’s depth, breadth, and gain. The mobility of a section of the left ventricular (LV) wall was observed using 2D echocardiography.

M-mode echocardiography was used to assess the LV’s end-diastolic dimensions (LVDd, LVDs), cardiac output (CO), and stroke volume (SV), as well as the LV’s end-systolic dimensions (LVAWd, LVPWd) and thickness of the LV’s anterior and posterior walls (LVAWd, LVPWd). The ejection fraction (EF) was calculated from the LV fractional shortening (FS) and LV volumes at the end of cardiac diastole and systole (LVVd, LVVs) using the Vivid 7 system software: EF = (LVVd − VVs)/LVVs 100%, FS = (LVDd − VDs)/LVDd 100%.

Cine loops consisting of 300 still images were created digitally and archived for all perspectives. A seasoned sonographer, who was unaware of which mouse model was being used, conducted the subsequent offline analysis. The 2D quantification program was used to obtain the LV volumes and EF.

### Statistical analysis

Data from the flow cytometry were analyzed with FlowJo, while statistical analysis was performed using Microsoft Excel and GraphPad Prism. In this case, data are presented as mean ± SEM. Student *t*-test was used for 2-group comparisons. Differences were considered statistically significant at *P* < 0.05.

## Results

### Characterization of human amnion mesenchymal stem cells

Cultured human hAMSC-13 could adhere to plastic, grow as a bipolar spindle-shaped, and display a fibroblast-like morphology (Fig. 1a). Our use of real-time fluorescence quantitative polymerase chain reaction allowed us to confirm that hAMSCs were free of bacterial contamination (Fig. 1b) or mycoplasma (Fig. 1c). They were then tested for their immunophenotypic features and their ability to differentiate into distinct cell lineages. In addition, the phenotypes of hAMSCs used in this study were confirmed by identifying MSC-specific surfaces biomarker by flow cytometry. The cells were discovered to express CD44, CD90, CD73, and CD105 while being deficient in HLA-DP/DQ/DR, CD11b, CD34, CD19, and CD45 (Fig. 1d). The ability of hAMSCs to develop into osteoblasts, chondrocytes, and adipocytes was also shown (Fig. 1e–g). Altogether, the results confirmed that the isolated hAMSCs could indeed be applied for downstream experiments.

### Establishment of age-related subclinical hypothyroidism mouse model

AR-SCH models were set up using female C57BL/6 mice as already described. With 20-month-old C57BL/6 mice considered to be equivalent to a human age of about 60 years, the animals were raised until they reached 20-month-old. Additionally, female mice aged 12 weeks were used as a young control (normal). Serum FT4 and TSH levels were measured in AR-SCH models to further assess model viability. While the two groups were not different in terms of FT4 levels (Fig. 2a), TSH expression was, on the other hand, significantly lower for the AR-SCH model in comparison with the control (*P* < 0.05) (Fig. 2b). Images of the thyroid gland (Fig. 2c–d), thyroid peroxidase antibodies (TPO-Ab) based on fluorescent staining (Fig. 2d–f'') and western blotting (Fig. 2g–h) are also provided.

Immunofluorescence staining showed that TPO-Ab expression in AR-SCH mice was higher than that in young control mice. Similarly, after extracting proteins from the thyroid tissues of the two groups of mice for western blotting, the results showed that TPO-Ab was highly expressed in AR-SCH mice (*P* < 0.05).

### hAMSC transplantation and in vivo tracing

After assigning the AR-SCH mice to treatment and control groups, the animals from each group were given injections as described earlier (Fig. 3a). However, since the thyroid gland has a small structure, fluorescent staining was not suitable for detecting STEM121 expression. After injection, hAMSCs were detected in thyroid by real-time fluorescence quantitative polymerase chain reaction (Fig. 3b). Futher, through the use of immunofluorescence, we were able to determine that the expression of human-specific gene STEM121 was observed in the liver and heart (SFig.1A).

### hAMSC-induced changes in hormone levels of AR-SCH mice

Thyroid function in SCH mice after hAMSC transplantation was evaluated by measuring circulating levels of thyroid hormones in the serum. There were no significant differences in FT4 levels between the groups before hAMSC injection (Fig. 3c), but the TSH level was significantly lower in the hAMSC group compared with the HAS group as from day 10 onwards (*P* < 0.01, Fig. 3d). To further detect changes in thyroid functions, the expression levels of serum thyroglobulin antibody (TGAb), thyroid microsomal antibody (TMAb), and thyroid peroxidase antibody (TPO-Ab) were determined. The results showed an obvious change in TPO-Ab which reached its lowest value on day 15 post-hAMSC injection (*P* < 0.01, Fig. 3e). Similarly, TGAb in the hAMSC group decreased as from day 10, with obvious differences between the two groups on days 10 and 15 (*P* < 0.05, Fig. 3f). However, the change in TMAb for the hAMSC group was not evident compared with that of the HAS group (Fig. 3g). Serum TGAb and TMAb represent two specific thyroid autoantibodies whose levels increase in autoimmune thyroid diseases. Indeed, TGAb is a specific diagnostic index of chronic lymphocytic thyroiditis, and its level is often significantly increased. We speculated even though the change in TMAb was not as significant, it would probably have been noticeable had the experiment been prolonged.

### Thyroid lesions and lymphoid infiltration

After administering the specified medications to the designated groups of model mice, the thyroid glands were excised and H&E stained. Visible signs of damage and atrophy in the thyroid follicles of the model mice were present, as was a thin colloid in the acinar cavity, demonstrating the infiltration of interstitial lymphoid cells. HAMSC therapy, on the other hand, reduced thyroid lesions and enhanced tissue integrity (Fig. 3h–o). Therefore, the findings provided evidence that hAMSC transplantation might lessen thyroid lesions and lymphoid infiltration.

Additionally, hepatic steatosis was significantly reduced in a visual analysis of liver and heart samples taken 5, 10, 15, and 20 days after hAMSC treatment. Following the treatment, the fat cells in the liver were also lower in numbers compared with the control (SFig.2A), while not many changes occurred for cardiomyocytes (SFig.2B).

### hAMSC transplantation positively changed lipid dysregulation in AR-SCH mice

Serum investigations showed hAMSC transplantation reduced lipid dysregulation. Serum levels of TC, TG, HDL-C, and LDL-C were analyzed to compare how hAMSCs altered AR-SCH mice lipid metabolism. The therapy group had lower LDL-C, TG, and T-CHO values than the HSA group. LDL-C dropped most on days 15 and 20, with a significant difference on day 20 (*P* < 0.05, SFig.2D). For TG and T-CHO, they was also a downward trend after hAMSC treatment, but there was no statistical difference (SFig.2E and F). In the end, no significant change in HDL-C was found (SFig.2C).

### hAMSC-induced transplantation to positively change heart function in AR-SCH mice

Our prior studies showed that injecting 300 μl of fluid into transplanted mouse hearts had no discernible adverse effects ^[20]^. To further evaluate the cardioprotective efficacy of hAMSC-injected AR-SCH mice, echocardiography revealed that left ventricular ejection fraction (LVEF), left ventricular fraction shortening (LVFS), cardiac output (CO), and stroke volume (SV) were enhanced in the hAMSC groups compared with the HSA group (SFig.3A). Notably, hAMSC treatment resulted in a higher LVEF (SFig.3B) and LVFS (SFig.3C) than HSA, but the results were not statistically significant. Similarly, we found that the AMSC group had higher CO (SFig.3D) and SV (SFig.3E) levels in day 5, 10, and 20 than the HSA groups, but the results were not statistically significant either.

### hAMSC adjustment Th1/Th2/Th17 cells and Treg cell percentage in vitro

IFN-γ, IL-4, IL-17 (Fig. 4a–d), and CD25 + CD125 + (Fig. 5a–c) cell secretion were all assessed by flow cytometry to ascertain if AMSC transplantation had an impact on cytokine release. The result showed that the IFN-γ (*P* < 0.01, Fig. 4e) and CD25 + CD125 + (*P* < 0.01, Fig. 5d) level of hAMSC group was significantly increased compared to the HSA group. Meanwhile, both IL-4 (*P* < 0.01, Fig. 4f) and IL-17 (Fig. 4g) results were significantly reduced in the hAMSC group than those in HAS group. Interestingly, in every groups, the levels of cytokines were reversed following hAMS implantation, with the highest change being seen at 15 days. The IFN-γ levels and CD25 + CD125 + cells rise after hAMSC transplantation with a maximum rise observed at 15 days after treatment. The level of IL-4 in hAMSC group decreased significantly after exposure. IL-17 slightly decreased no statistical significance; however, they were still lower than the HSA groups.

### HAMSC transplantation inhibited the apoptosis of thyroid tissue in the AR-SCH mice

The link between the immune-modulatory effects of hAMSC treatment and the apoptosis signaling pathway in SCH was investigated by examining the expression levels of cell apoptotic proteins in thyroid, liver, and heart. TUNEL analysis of the cells revealed that there was a substantial reduction in the number of TUNEL-staining cells in the hAMSC group compared to the untreated group on day 15, 20 after hAMSC injection, showing a reduction in thyroid apoptosis (*P* < 0.05, Fig. 6a–i). The similar observations were made on hepatocytes (SFig.4A) and cardiomyocytes (SFig.4C); on the 15 days after hAMSC injection, there was a significant difference in liver cell (*P* < 0.05). By using western blotting, we analyzed the relative expression of proteins implicated in the apoptotic signaling pathway in thyroid tissues (Fig. 6j). We found that the expression of caspase 3 (Fig. 6k) and BAX (Fig. 6m) was decreased. And, the expression of Bcl 2 in thyroid observed has statistical difference on day 15 (Fig. 6l).

## Discussion

Thyroid autoimmunity and thyroid dysfunction become common with the aging process. For instance, several small-scale studies have found that TSH and anti-thyroid antibody levels increase with age, especially in female patients (Canaris et al. 2000; Hollowell et al. 2002). Currently, the routine clinical treatment for SCH mainly includes levothyroxine (Vanderpump et al. 1995). However, the efficacy of levothyroxine in reducing mortality and morbidity or even in improving quality of life in elderly patients with hypothyroidism has been controversial (Effraimidis et al. 2021). Furthermore, there is a risk of overtreatment when using L-T4, especially in older adults (aged from 78 to 80) (Vaisman et al. 2013). Indeed, among all patients taking L-T4, 5% to 24% develop iatrogenic thyrotoxicosis, with the proportion rising to as high as 41% in the elderly (Somwaru et al. 2009). Therefore, a new method to treat SCH is urgently needed.

Mesenchymal stem cells (MSCs) represent one type of adult stem cells derived from the mesoderm, and they are the most effective ones for the treatment of autoimmune diseases (Catalina et al. 2007). Indeed, through their unique immunomodulatory properties, they can significantly inhibit the activation of T lymphocytes (Silini et al. 2017). Similarly, hAMSCs are a group of mesenchymal stem cells derived from human amniotic membrane, and in addition to the ease with which they can be collected, they are also characterized by a strong proliferative ability as well as low antigenicity (Miki 2018). So far, HAMSCs have been used to study various refractory diseases such as type 2 diabetes (Zang et al. 2022), inflammatory bowel disease (Soontararak et al. 2018), and premature ovarian failure (Lu et al. 2019). As such, they are emerging as a promising form of treatment for different human refractory diseases.

In this study, an AR-SCH mouse model was established to investigate the therapeutic effect of hAMSCs on SCH and the underlying mechanism of action. It was shown that hAMSCs could migrate to the thyroid, heart, and liver where they effectively repaired thyroid dysfunction while improving blood lipid levels and cardiac function. More importantly, hAMSCs inhibited apoptosis in aged SCH mice by reducing inflammatory response and modulating cellular immunity.

A small amount of cell colonization was observed in the thyroid tissue of mice 5 days after injection of hAMSCs, with the cells also detected in the liver and heart. On day 10 after hAMSC treatment, a decreasing trend in TSH was then noted, as usually, the “homing” of exogenous MSCs to the site of injury is limited and does not last for a long time (Montanari et al. 2015). Due to T4’s plasma half-life of 7 days and thyroid-binding globulin’s half-life of 5 to 7 days (van et al. 2017), a booster hAMSc injection was then made on day 10. Consequently, TSH improvement was more pronounced on day 15, while TPO-Ab and TgAB displayed an obvious decrease on day 15 after hAMSC injection.

Histological changes of the thyroid gland in aged SCH mice showed follicular epithelial hyperplasia, follicular atrophy and destruction, and interstitial edema as well as different degrees of inflammation-mediated cell infiltration. After treatment, the proliferation of follicular epithelium in the thyroid tissue of mice was improved, the follicular cavity was filled with homologous glia, and the surrounding inflammatory cell infiltration was reduced.

SCH is associated with a range of events (left ventricular systolic and diastolic dysfunction and impaired vasodilation) which increase cardiovascular abnormalities (Sue et al. 2020). Cardiac ultrasound was performed in the experimental mice to assess whether hAMSCs could improve cardiac function. Results of M-mode echocardiography showed that ejection fraction (EF), fractional shortening (FS), CO, and SV increased, while all other measures of cardiac function remained unchanged. In addition, there were no significant differences in the pathological sections of heart tissues between the groups.

Hypothyroidism is one of the most common secondary causes of dyslipidemia (elevated levels of LDL-C and TG), and hence, screening for thyroid function is often recommended in patients with hypercholesterolemia (Zhou et al. 2020). In our current study, treatment with hAMSCs improved dyslipidemia in aged SCH mice, including reductions in LDL-C and TG levels. Histological staining of the liver also showed an improvement in hepatocyte steatosis with increasing days after treatment, but lower hepatic adipocytes were noted compared with the control group.

SCH is mainly caused by autoimmune thyroid diseases (AITD), a group of T cell-mediated, specific autoimmune diseases of the thyroid tissue (Yoo and Chung et al. 2021). The main characteristics of this disease are the infiltration of a large number of lymphocytes in the gland as well as the formation of specific thyroid autoantibodies which eventually lead to the destruction of glandular tissue and glandular hypofunction (Weetman 2020). However, the direct cause of the autoimmune attack on the thyroid has not been clarified although currently; it is believed that an autoimmune reaction, possibly related to an imbalance of Th1/Th2 and upregulated Th1 immune response (Nauta and Fibbe 2007), could be the main pathogenetic mechanism. Th1 cells mainly secrete interleukin-2 (IL-2), interferon-γ (IFN-γ), and tumor necrosis factor-β (TNF-β), while Th2 cells secrete interleukin-4 (IL-4), interleukin-6 (IL-6), and interleukin-10 (IL-10) (Mosmann et al. 1996). Furthermore, Th17 cells are considered to be important pathogenic factors in Th1-dependent autoimmune diseases (Bedoya et al. 2013). Hence, the pathogenesis of AITD is complex, especially since it is closely related to the immune dysfunction of thyroid tissues. Consequently, regulating and restoring the body’s autoimmune function is at the core of AITD’s treatment.

The results showed that after hAMSC transplantation, the expression of IL-4 and IL-17 decreased, while that of IFN-γ increased significantly in the hAMSC group compared with the control group. At the same time, the level of Treg cells also increased with increasing days after treatment. Overall, these results were consistent with previous reports. It was also found that the immune regulation of mice in the experimental group changed on the 5th day after hAMSC transplantation, with improvement in Th1, Th2, and Treg levels being most evident on the 15th day. Thus, it was clear that the booster injection of hAMSCs could enhance the regulatory functions of immune cells.

It has been suggested that cytokines are key factors that initiate immune disorders in thyroid tissues. In addition, many studies have confirmed that Fas-mediated apoptosis was involved in the pathogenesis of AITD (Strasser et al. 2009). For instance, Bossowski A et al. found that the destruction of thyroid follicular cells in Hashimoto’s thyroiditis patients was related to the Fas-mediated apoptosis pathway (Bossowski et al. 2006). In this case, infiltrating lymphocytes exert cytotoxicity on nearby thyroid cells via FasL paracrine to promote apoptosis (Maruoka et al. 2004).

To further elucidate the effects of hAMSCs on thyroid function, TUNEL assay was employed to detect the apoptosis level of thyroid cells, hepatocytes, and cardiomyocytes. The results showed that with increasing time since the hAMSC injections, the apoptosis level of thyroid cells, hepatocytes, and cardiomyocytes decreased. Western blot further indicated that the expression of BAX and caspase3 decreased, while the level of Bcl2 increased. Altogether, the findings suggested that hAMSC transplantation could improve thyroid function by inhibiting the mitochondrial apoptosis pathway of thyroid cells.

### Supplementary Information

Below is the link to the electronic supplementary material.

Supplementary file1 (TIF 11054 KB)

Supplementary file2 (TIF 29204 KB)

Supplementary file3 (TIF 10257 KB)

Supplementary file4 (TIF 19168 KB)

## Data Availability

The data presented in this study are available on request from the corresponding author.
