# Supplementary figures and images for: Human amnion-derived mesenchymal stem cells improve subclinical hypothyroidism by immunocompetence mediating apoptosis inhibition on thyroid cells in aged mice
Source: Cell Tissue Res. 2023 Aug 12;394(2):309–23. doi: 10.1007/s00441-023-03822-1 (PMC10638193; doi:10.1007/s00441-023-03822-1)

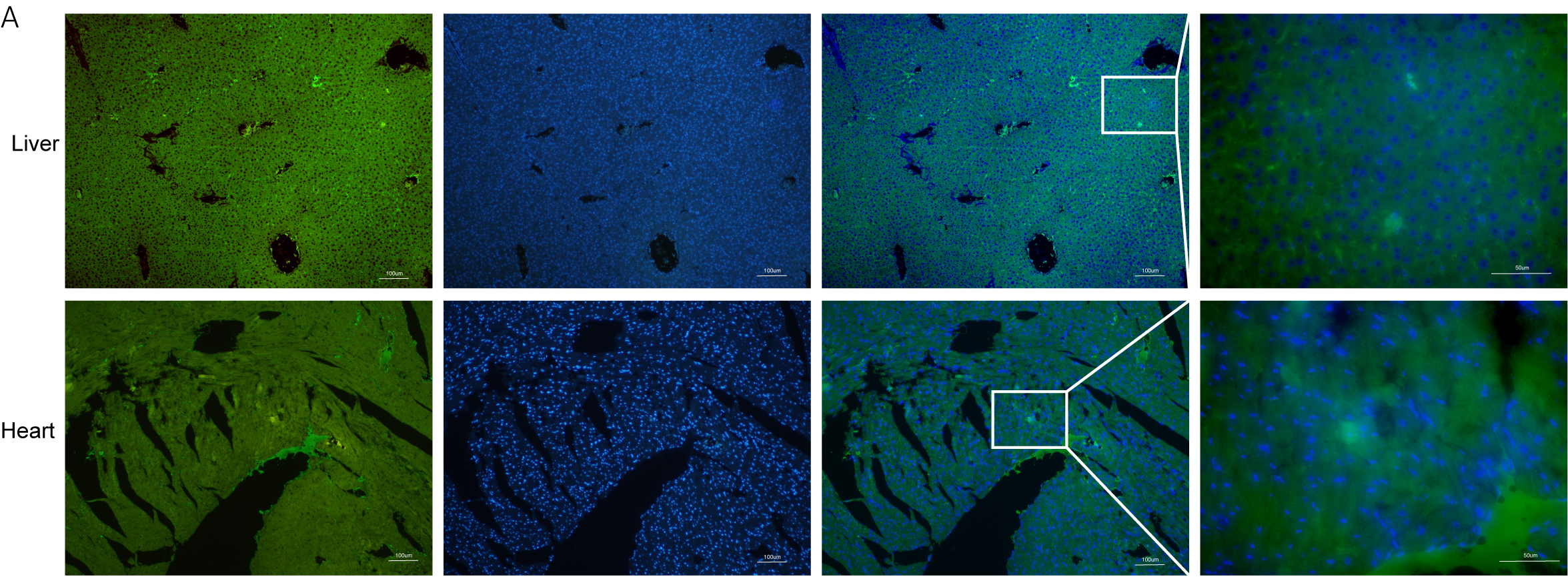

Supplement: Supplementary file 1 — Supplementary file1 (TIF 11054 KB) [file 441_2023_3822_MOESM1_ESM.tif]

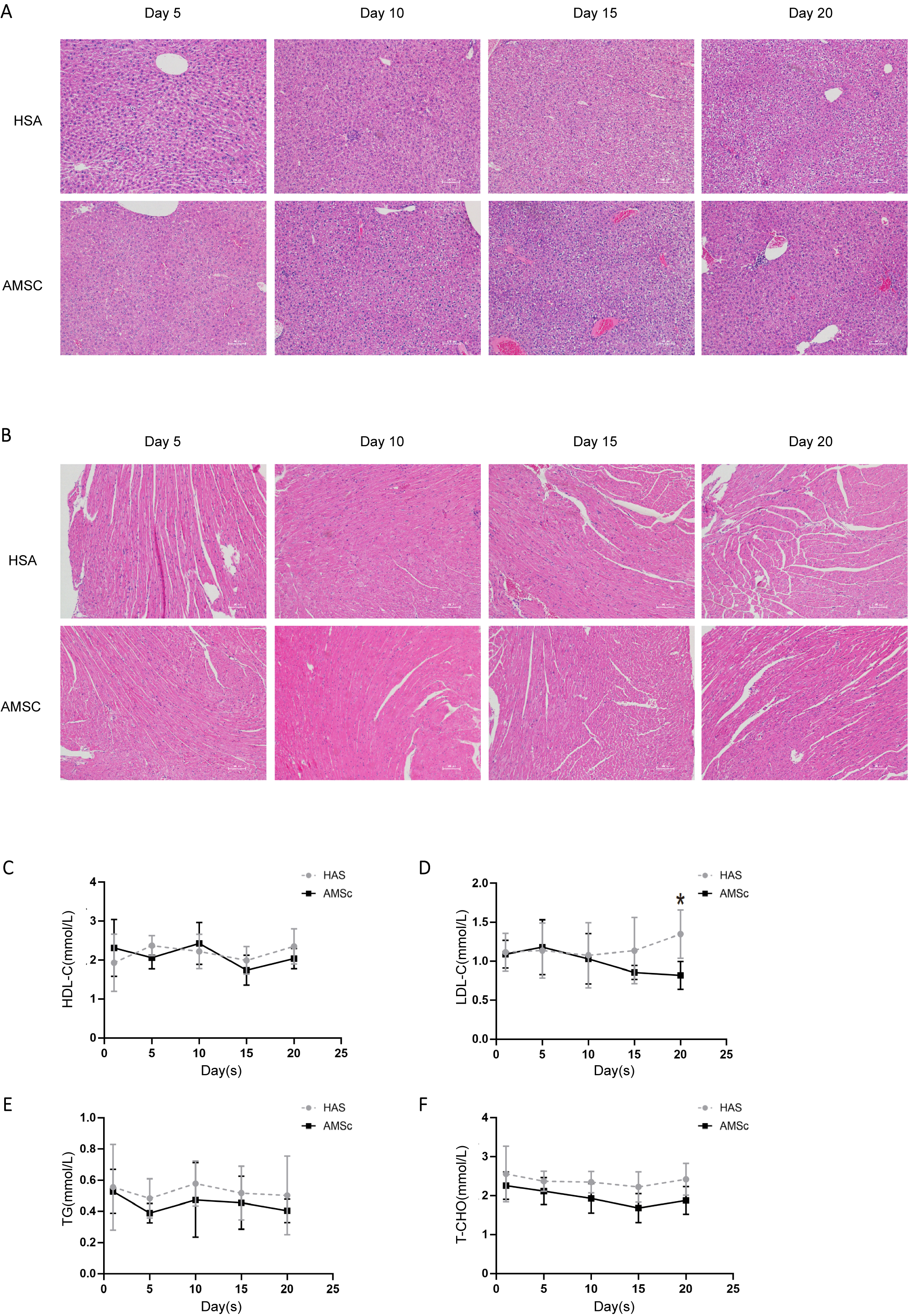

Supplement: Supplementary file 2 — Supplementary file2 (TIF 29204 KB) [file 441_2023_3822_MOESM2_ESM.tif]

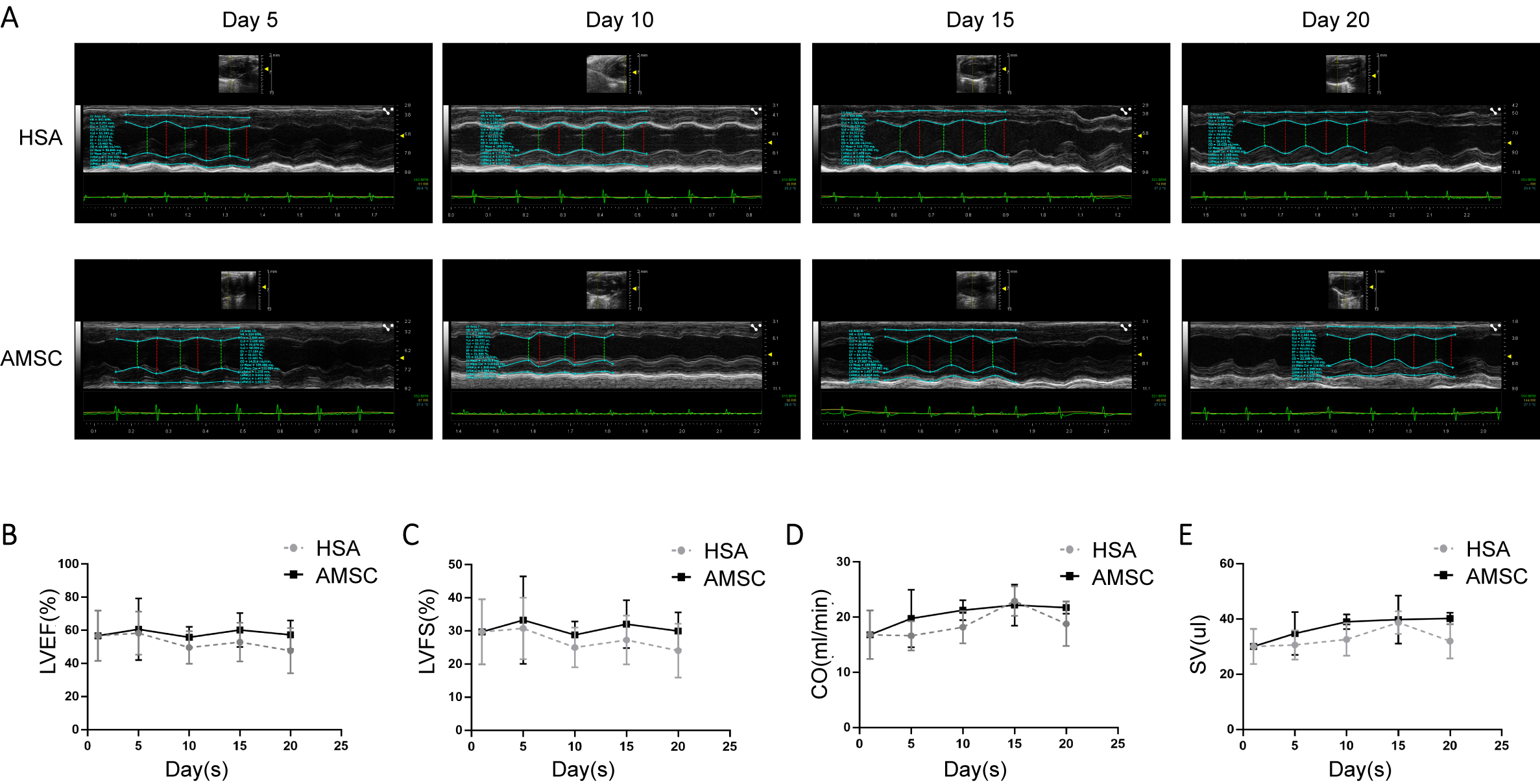

Supplement: Supplementary file 3 — Supplementary file3 (TIF 10257 KB) [file 441_2023_3822_MOESM3_ESM.tif]

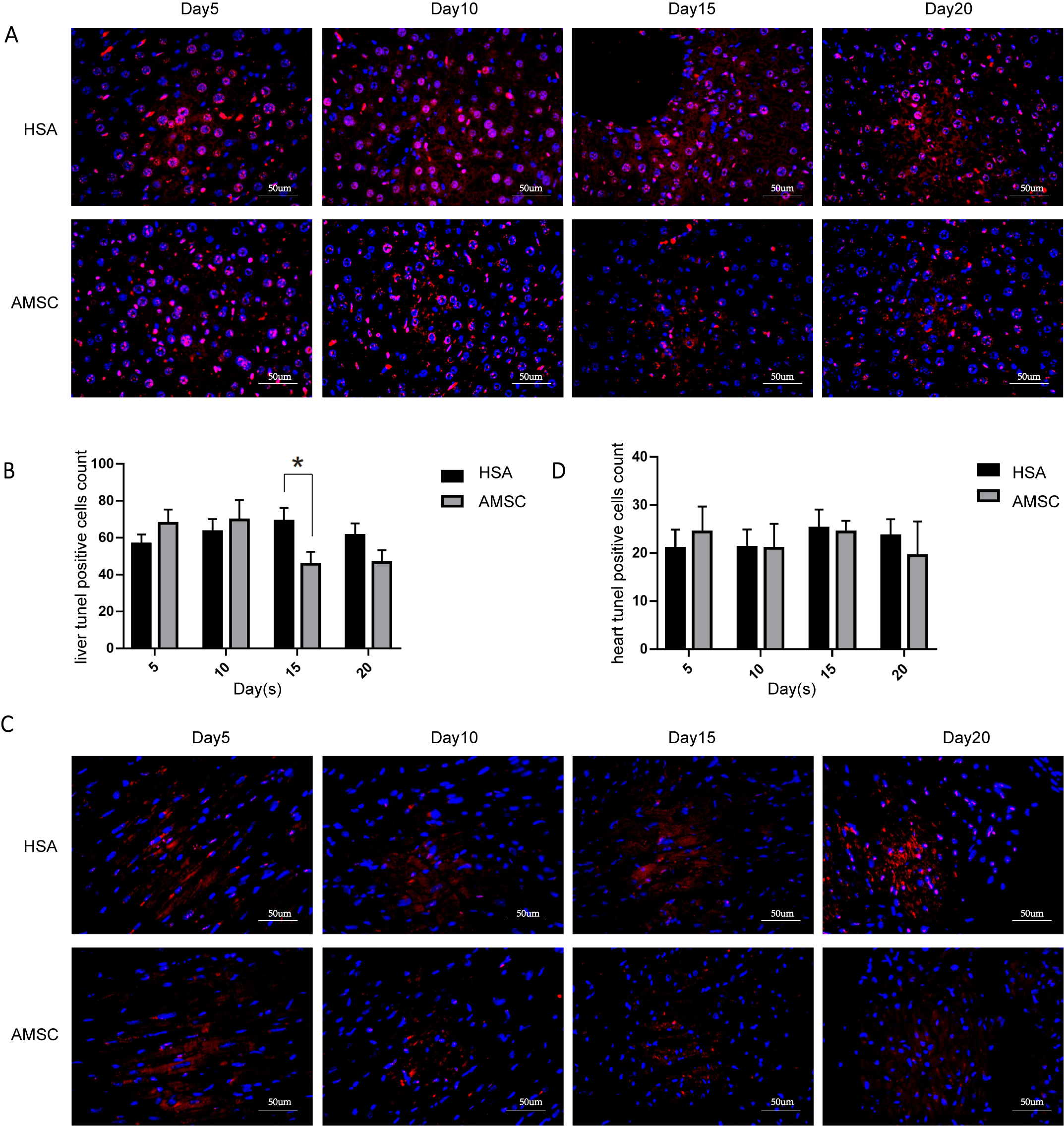

Supplement: Supplementary file 4 — Supplementary file4 (TIF 19168 KB) [file 441_2023_3822_MOESM4_ESM.tif]
